# Efficacy and acceptability of an “App on sick newborn care” in physicians from newborn units

**DOI:** 10.1186/s12909-016-0579-3

**Published:** 2016-03-08

**Authors:** V. Prakash, Anu Thukral, M. Jeeva Sankar, Ramesh K. Agarwal, Vinod K. Paul, Ashok K. Deorari

**Affiliations:** Department of Pediatrics, All India Institute of Medical Sciences, New Delhi, India

**Keywords:** Mobile-applications, Apps, Special newborn care units, Smart phones

## Abstract

**Background:**

There has been an increased emphasis on institutional births, and thus an increasing clinical work load for health care professionals in the recent past. Hence, continuing education, training, ongoing supervision, and mentorship of health care professionals working in these health facilities with easy access to guidelines in a cost effective manner has become a challenging task. With the increased emphasis on institutional births, and an increasing clinical work load, continuing education and training of health care professional managing these health facilities, their ongoing supervision, mentorship, with ready availability of guidelines in a cost effective manner becomes imperative and is a challenging task. Training opportunities can be linked to mobile electronic devices and ‘Apps’ to improve the care of seriously ill newborn. The aim of this study was to evaluate the efficacy of an innovative point of care tool- Android based App- ‘AIIMS-WHO CC STPs’ on the knowledge, skill scores, and satisfaction among Special Newborn Care Unit (SNCU) physicians managing sick neonates.

**Methods:**

The baseline knowledge and skill scores of pediatricians working in SNCUs in the state of Tamil Nadu, India (*n* = 32) were assessed by 25 multiple choice questions (MCQs) and by five Objective Structured Clinical Examination (OSCE) skill stations. The training was conducted in a single-day workshop using the app on four modules followed by post-training assessment of knowledge and skill scores after 3 weeks using the same. The satisfaction was assessed by mixed method approach using Likert’s scale and focus group discussion (FGD) after 3 weeks.

**Results:**

The mean knowledge scores [19.4 (2.6) *vs.* 10.7 (3.2); maximum marks (MM) 25, mean difference 8.7 (95 % CI 7.6 to 9.9)], and the composite mean skill scores [55.2 (5.8) and 42 (6.2), MM 75, mean difference 13.2 (95 % CI 10.4 to 15.9)] improved after training. The median (IQR) satisfaction score with the course was 4 (4 to 5) (Likert’s scale). Focus group discussion revealed that the physicians were overall satisfied using the device. They expressed overall satisfaction on the teaching methodology using wall charts, simulators, and device.

**Conclusion:**

Training SNCU physicians on Android based App- ‘AIIMS-WHO CC STPs’ improved their knowledge and skills. This app may have a potential role as a supplement to other modalities in training doctors for improving newborn care.

## Background

Every year worldwide, nearly 2.76 million newborns die from largely preventable causes with nearly 1 million dying on the first day itself [1]. About 0.75 million neonates die every year in India; the highest for any country in the world [1]. In India, the share of neonatal mortality to the under-five mortality has increased over time from 41 % in 1990 to 56 % in 2012 as against a worldwide contribution of 44 % [2, 3].

It is being increasingly recognized that a two-third reduction in childhood mortality from 1990 to 2015 (Millennium Developmental Goal 4) can only be achieved by a reduction in neonatal deaths [4]. In India, an important step in this direction has been the emphasis on institutional deliveries and strengthening of “Facility based newborn care” in the public health system, which envisages a nationwide creation of special newborn care units (SNCUs) at district hospitals in addition to newborn Care Corners (NBCC) at every point of child birth, and newborn stabilization units (NBSU) [5, 6].

With the increase emphasis on institutional births in India and increasing clinical work load, continuing education, and training of health care professional managing these health facilities becomes imperative. In addition, bedside availability of clinical guidelines and the ongoing mentorship of health care professionals in a cost effective manner is a challenging task [7, 8]. Linking the training opportunities of these health care professionals to mobile electronic devices, and ‘Apps’ to improve the care of seriously ill newborn seems a possible way forward.

Using a smartphone ‘app’ at remote places as clinical management tool may be a good supplement to conventional training methodology (teacher-driven teaching with lectures, textbooks or presentations) [9–11]. Smart phone ‘app’ is an application that which provides immediate electronic access in multiple fields. In medical field, it may help keep track of appointments, aid collection of real time data [12]; and helps and assess nutrition environment [13] in addition to multiple other uses. App installations have multiplied the market of smartphone immensely, and it is growing rapidly [9–11, 14, 15].

Amongst the other available medical apps on smart phones, the algorithmic management of common conditions in sick neonates has been created as point of care tool on Android Google play for health-care professionals by the World Health Organization Collaborating Centre (WHO-CC) for Training and Research in Newborn Care, All India Institute of Medical Sciences (AIIMS), New Delhi. This has already been tested for content reliability and validity at WHO-CC.

This ‘App’ in addition to being user-friendly, has the advantage of easy, immediate availability as a point of care tool, and gives an opportunity to learn skills related to procedures and equipment. The efficacy and acceptability of this ‘App’ has already been evaluated among nursing college students [16]. This study was planned to evaluate its efficacy in the actual target group (Medical Officers) who are handling the sick newborns in SNCUs.

## Methods

### Subject and setting

The study was conducted from March 2014 to February 2015. Thirty-two medical officers treating neonates in Special Newborn Care Units from 25 districts of Tamil Nadu participated in the study conducted at the Institute of Child Health (ICH), Chennai, Tamil Nadu, India. These pediatricians were recruited from the SNCUs attached to district hospitals, and had a postgraduate degree (MD or Diploma in Child Health) in Pediatrics. The participants were selected by the state Integrated Management of Neonatal and Childhood Illness (IMNCI) coordinator based on the availability, and willingness of the candidates to participate (which all participants expressed).

The workshop was conducted by instructors from the All India Institute of Medical Sciences (AIIMS) at the Institute of Child Health (ICH), Chennai, Tamil Nadu, India. The Institute’s ethics committee approved the study. Permission was obtained from the Health and Family Welfare Department of Tamil Nadu to conduct the study. An informed written consent was obtained from all the participants. A separate informed consent for the FGD (and recording its proceedings) was also obtained from each participant. None of the medical officers had used this ‘App’ before participation in the study.

### Training content and methodology

The methods and tools were pilot tested and peer reviewed by three independent experts. The training of participants on four modules, namely- (1) Hypothermia, (2) Seizures, (3) Shock, and (4) Feeding of the low birth weight (Table 1) was done in a one day workshop. The participants were divided into four groups, and a facilitator for 8 participants at four workstations taught each module. The facilitators were neonatologists with over 10 years of experience in teaching and training. The facilitators at each workstation demonstrated the algorithmic management of the respective clinical conditions on the “App” on the tablet [bigger screen (9 inch) than smartphone], followed by interactive discussions. The facilitators used preloaded videos of respective procedures with the demonstration. The participants were later given the opportunity of ‘hands-on’ experience for using the App loaded in the tablet devices. In addition, those who had their own smartphones/tablets were assisted to download the ‘App’ in their respective devices. The participants were shown apps on all newborn conditions (total = 12) but practice opportunity was provided for only four selected modules mentioned above. An independent team consisting of two senior neonatologists supervised the execution of the training workshop.

**Table 1:** Content of the topics used in module

Topic	Core Content
Hypothermia	• Approach to a neonate with hypothermia
	• Video on temperature recording
Seizures	• Approach to a neonate with seizures
	• Common causes of seizures
	• Differences between seizures and jitteriness
	• Protocol for administration of common anticonvulsants
	• Video on blood glucose estimation
Shock	• Approach to a neonate with shock
	• Monitoring of a baby with neonatal shock
	• Video on insertion and fixation of intravenous line
Feeding of the low birth weight and sick neonate	• Choosing the initial feeding method and stepwise approach of increasing the feed volume
	• Video on alternative feeding method
	• Video on breast milk expression

### Outcome assessment

The primary outcome of the study was assessment of improvement in knowledge and skills. All the participants were given 25 multiple-choice questions (MCQs) on the clinical modules (shock, hypothermia, seizures, intravenous fluid therapy for newborn, and feeding of low birth weight and sick babies) prior to the training workshop to assess their baseline knowledge. The MCQs were written and then peer reviewed by two independent content experts, and process experts (available at. http://www.newbornwhocc.org/MCQ_and_OSCE_Questions_Apps.pdf.).

The assessment of baseline skill scores was performed using five Objective Structured Clinical Examination (OSCE) skill stations (management of shock, demonstration of alternative method of feeding, expression of breast milk, preparation of drugs related to the four selected modules (phenobarbitone), and measurement of axillary temperature) (available at http://www.newbornwhocc.org/MCQ_and_OSCE_Questions_Apps.pdf.). The knowledge and skills of the participants were again evaluated after the training workshop (after 3 weeks).

The secondary outcome of this study was to evaluate the perception of participants towards the workshop methodology, content, and the device, which was assessed by, mixed method approach using Likert’s scale (5 point scale; 1 = strongly disagree, 2 = disagree, 3 = neither agree nor disagree, 4 = agree, 5 = strongly agree), and focus group discussion (FGD). A Likert’s scale (20 questions) questionnaire on content relevance, teaching methodology, applicability, and perceived utility were administered at the end of the workshop.

FGD with a group of 12 participants (chosen randomly from 12 different SNCUs) was conducted in the follow-up session 3 weeks later. The questions included feedback on the training, performance of Apps with regards to their strengths and limitation, their user-friendliness, and feasibility of application as a bedside tool, the potential obstacles anticipated, and avenues to overcome these obstacles, and the ways to improve the training. These discussions were analyzed for common themes. We did not do a specific validity or reliability assessment of these questions. These focus groups used some general probes and not a specific questionnaire including: “Would you like to explain further?” “Would you give me an example of what are you trying to tell?”; “Would you like to say more?”; and “I do not understand”. All the FGD proceedings were first recorded in duplicate, and then transcripts were compiled for qualitative analysis.

### Data collection

Each participant was assigned a unique number. The main investigator (VP) and the facilitators (AKD, AT) assessed data set on the MCQ scores on pretest, and post-test, and the OSCE stations scores, independently. These scores were entered analyzed using Microsoft Excel®.

### Data analysis

Analysis was done using Stata 11.2 (Stata Corp, College station, TX, USA). Data were presented as mean (SD) or median (IQR) or frequency (%). Means and standard deviations for knowledge and skill scores were computed. Continuous variables were compared using paired ‘t’ test and categorical variables were analyzed using McNemar test. *P* value of < 0.05 was taken as significant. We assumed that atleast 75 % of marks must be scored by the participants in MCQs and OSCE stations for passing in knowledge and skill assessment individually. The satisfaction of the learners is expressed as median (IQR range).

Two authors using constant comparison method (Glaser and Strauss) [17] analyzed the transcriptions of data from the FGD independently. We used method of triangulation for the purpose of validation i.e., we corroborated data from other sources (Likert’s scale and informal discussion held with the participants).

We assumed that the baseline scores of the SNCU’s doctors to be 18 ± 4 of a maximum possible score of 25, and the expected knowledge scores after the training to be 20 ± 4. For a power of 90 %, the estimated sample size was 27 each for pre and post training knowledge assessment. Assuming some attrition loss of trainees during the follow-up, we enrolled 32 SNCU doctors.

## Results

Thirty-two pediatricians [age, 36.2 (7.4) years] from 25 districts of Tamil Nadu participated in the study. Out of them majority (29, 90.6 %) had a post graduate diploma in child health remaining had a post graduate degree in pediatrics. The clinical experience (years of treating neonates), and work experience in SNCU’s were was 2.0 (1.5, 5) years and 1.6 (0.6, 2) years respectively (Table 2). Five participants did not report to for the post-test because of dengue outbreak in their districts. Thirty participants (93.8 %) were comfortable in using Internet and had smart phones (Table 2).

**Table 2:** Demographic details and clinical experience of the participant

Variables	*n* = 32
Age in years	36.2 (7.4)
Male	24 (75 %)
Qualification	
• Diploma in Child health (D Ch.)	29 (90.6 %)
• Diploma in National Board (Pediatrics)	1 (3.1 %)
• M.D. in Pediatrics	2 (6.3 %)
Clinical experience of years in treating Newborn	2 (1.5–5)
Years of working in Special Newborn Care Units	1.6 (0.6–2)
Already having smart phone	30 (93.8 %)
Comfortable in using Internet	30 (93.8 %)
Already having medical apps	23 (71.9 %)
Availability of Internet access at SNCU	21 (65.6 %)

Values expressed as mean (±SD), number (%), median (IQR)

**Fig. 1:**
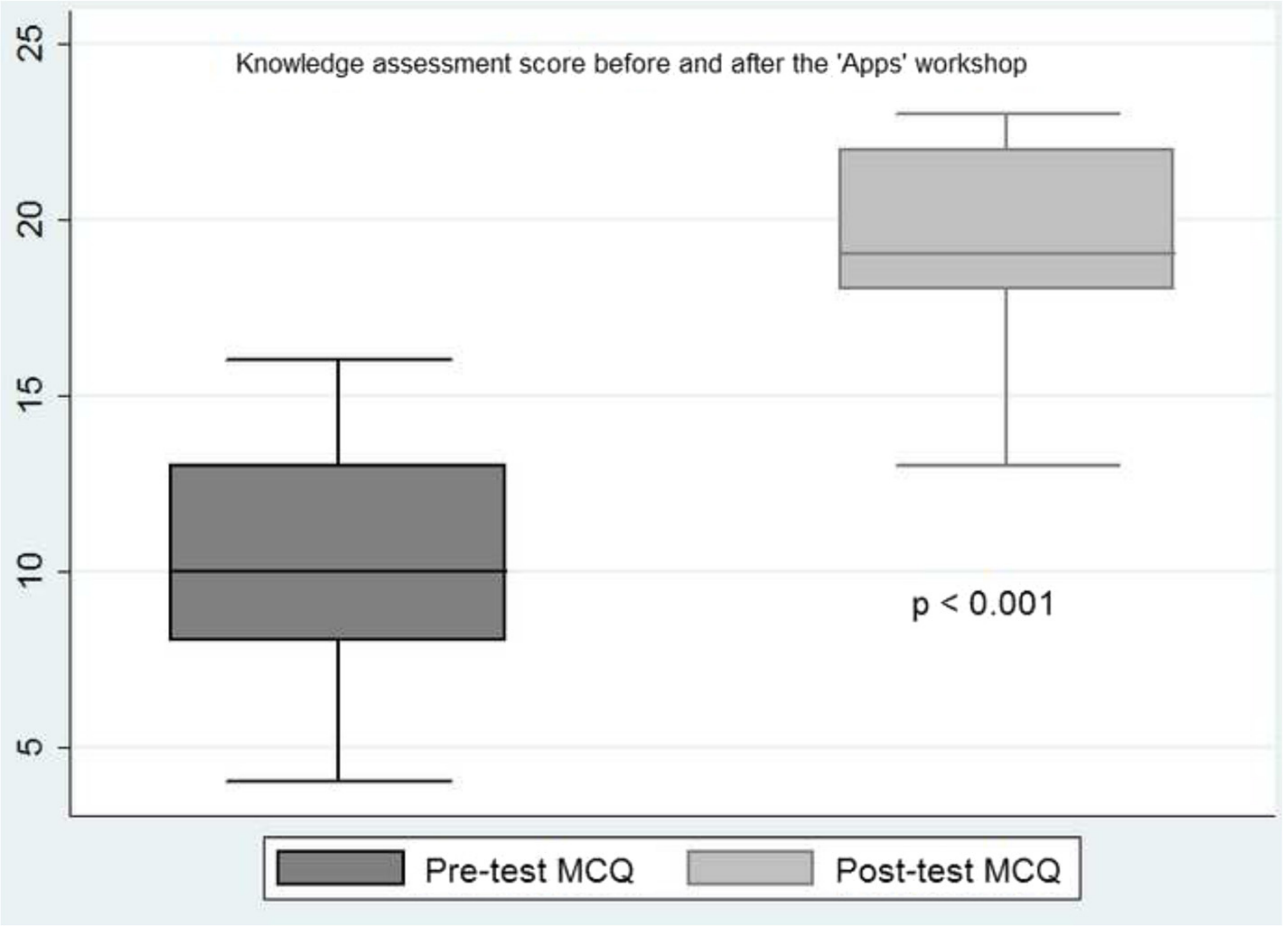
Marks scored in knowledge assessment before and after the workshop (Maximum Marks 25)

**Fig. 2:**
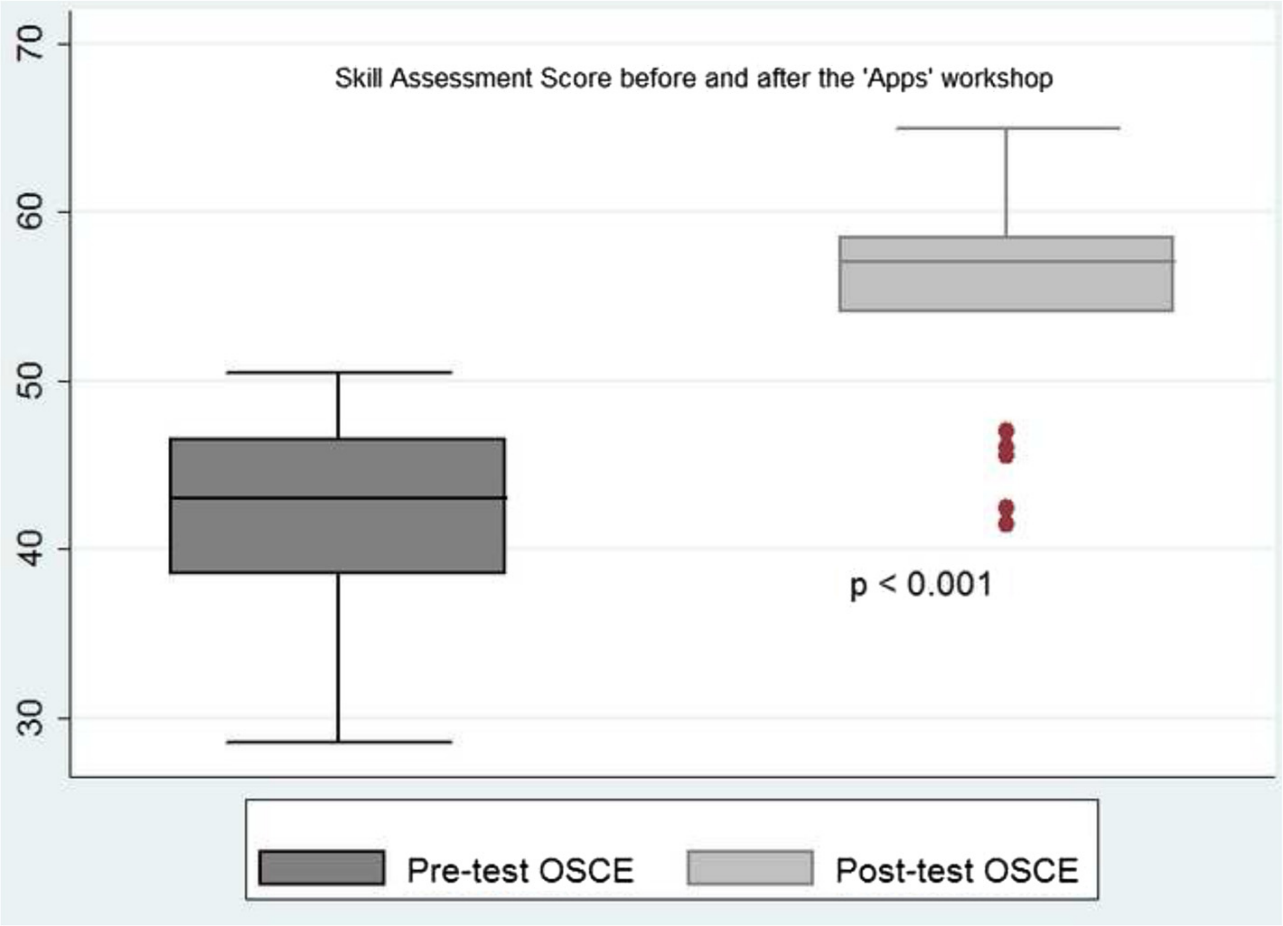
Marks scored in skill assessment before and after the workshop (Maximum marks 75)

There was a significant improvement in the knowledge scores [pre- and the post-test scores, 10.7 (3.2) *vs.* 19.4 (2.6), mean difference 8.7, 95 % CI (7.6 to 9.9)], and the composite skill scores of the participants [pre- and the post-test scores, 42 (6.2) and 55.2 (5.8), mean difference 13.2, 95 % CI 10.4 to 15.9] (Table 3, Figs. 1 and 2)

**Table 3:** Knowledge and skill scores

Outcome variables	Pretest *n* = 27	Posttest *n* = 27	Mean difference 95 % CI	*p* value
Knowledge scores^a^	10.7 (3.2)	19.4 (2.6)	8.7 (7.6–9.9)	<.001
Skill scores: Composite Scores^b^	42 (6.2)	55.2 (5.8)	13.2 (10.4–15.9)	<.001
OSCE station 1 (Management of shock)	7.9 (2)	10.1 (1.4)	2.2 (1.3–3.1)	<.001
OSCE station 2 (Alternative feeding methods)	6.6 (2.8)	12 (3.0)	5.4 (4.1–6.8)	<.001
OSCE station 3 (Temperature recording)	10.5 (1.7)	11.9 (1.9)	1.5 (0.7–2.3)	<.001
OSCE station 4 (Expression of breast milk)	6.7 (2.1)	10.4 (2.3)	3.7 (2.5 to 4.8)	<.001
OSCE station 5 (Medication preparation)	10.2 (1.8)	10.7 (1.1)	0.4 (−0.5–1.3)	0.16

Values expressed as mean (±SD); ^a^Max score 25, ^b^Max score 75

All independent OSCE stations maximum score 15

Assuming that atleast 75 % of marks must be scored by the participants in MCQ‘s and OSCE stations for passing in knowledge and skill assessment, Only one-third (11/27; 34.4 %) of doctors passed both in knowledge and skill scores individually in the post-test. The pass percentage was higher in knowledge assessment (11/27; 74 %) when compared to skill assessment (composite score) pass percentage of (15/27; 55.6 %) in the post test scores. There was significant improvement in pass percentage among doctors in the posttest in all stations except the OSCE station for shock and drug preparation (Table 4).

**Table 4:** Doctors who obtained more than 75 % marks in knowledge and skill assessment

Outcome variable	Pre-Workshop *n* = 27	Post Workshop *n* = 27	*p* value
Pass in MCQ s and OSCE individually	0	11 (34.4 %)	<0.001
Pass in MCQs	0	20 (74 %)	<0.001
Pass in OSCE (composite score)	0	15 (55.6 %)	<0.001
Pass in OSCE-Management of Shock	1 (3.1 %)	3 (11.1 %)	0.3
Pass in OSCE –Alternative methods of feeding	2 (6.3 %)	20 (74 %)	<0.001
Pass in OSCE -Temperature recording	8 (25 %)	16 (59 %)	0.007
Pass in OSCE- Expression of breast milk	0	12 (44.4 %)	0.005
Pass in OSCE Drug preparation	11 (34 %)	5 (18.5 %)	0.29

Values are expressed as number (%)

The participants expressed overall satisfaction from the training (Table 5). In addition, all they felt empowered to be able to implement the techniques learnt, into day to day practice.

**Table 5:** Participants satisfaction from the training on Likert’s scale

Participants satisfaction scale	Likert’s scale
Flow diagram was explicit and clearly defined	4 (4–5)
PDF on STP was explicit and clearly defined	5 (4–5)
Procedures /skill videos was explicit and clearly defined	5 (4–5)
Overall content was explicit and clearly defined	4.5 (4–5)
Equipment demonstration was explicit	4 (4–5)
Training useful for professional activities	5 (4–5)
Workshop on APPs has increased your confidence to achieve personal objectives	5 (1–5)
Will you be able to implement the techniques learnt during the sessions	4 (4–5)
The session will help to put the knowledge gained into practice	4 (4–5)
You achieved overall satisfaction from the course	4 (4–5)
The videos and procedure related skills are related to the context and helpful	4 (4–5)
The application is easy to learn.	4 (4–5)
The application is interactive and user friendly	4 (4–5)
The content adds to your previous knowledge and understanding of the specific topic	4 (4–5)
The algorithm approach was very useful	4 (4–5)

*Note:* 1 = Strongly Disagree,2 = Disagree, 3 = Neither agree nor disagree, 4 = Agree, 5 = Strongly Agree

### Focus group discussion

During the FGD, the participants expressed that the ‘App’ was an innovative approach and using the flow charts were simple, useful, and it addressed their doubts. The list of apps ‘App’ covered all the common newborn conditions they come across in their work in the special newborn care units. They expressed that the ‘App’ met their expectation. Participants felt that the strengths of ‘App’ was it’s simple language, easy accessibility and structured approach in the management of newborn conditions. It was felt that the videos require Internet connection and if the ‘App’ was created with videos embedded so as to require only a one-time download, it would be more useful. In addition, it was felt that the ‘App’ is available only in Android and iOS platform, if the ‘App’ is supported by Windows; the accessibility would perhaps be greater and they could show the videos and protocols to nursing staff on the desktop since all SNCUs have computers.

The participants expressed that the monthly salary of nursing staff is between Rs 6000/- to Rs 8000/- (USD 100 to 150); hence the cost of smartphones may be limiting factor for its practical use by nursing professionals. They felt that protocol management of hypoglycemia was very good, and previously, they had not followed any guidelines. There was a felt need for guidelines and protocols for management of newborn jaundice to be expressed in tabular form. The MCQs made them understand helped reflect their level of knowledge before and after the workshop, were easy to understand but they felt that the number could be possibly increased with more case scenarios. They felt that workshop should be conducted over 2–3 days to enable them to cover all topics and related videos, and that the workshop should be made a regular biannual phenomenon. It was suggested by some personnel that if the protocols (Apps) were incorporated into Health Monitoring Information System in their state, then all SNCU Doctors would be obliged to follow the same protocol. Some quotes *ads verbatim from the FGD are* given in Panel 1.

## Discussion

Our study demonstrated a significant improvement in knowledge and composite skill scores of the trainees using the ‘AIIMS-WHO CC STPs’ app. The pass percentage in knowledge domain was 74 % when as compared to 55.6 % in the skill assessment. The participants felt confident after the training and were willing to use the ‘App’ in their SNCUs.

The utility of other apps in the field of medicine for monitoring in adult population in chronic disease [18–22], treatment [23, 24], emergency resuscitation [25, 26], and drug dosage calculation [27] have been previously reported. A study of apps for neonatal intubation has also shown improvement in the skills and knowledge scores [28]. Efficacy of the Android based App- ‘AIIMS-WHO CC STPs’ in sick newborn care has been reported earlier among the nursing students [13] but the present study was done among the physicians who are involved in the direct management of sick newborns at district hospital level and at most times have to manage the sick newborn single handed.

The present study has successfully demonstrated the efficacy and usefulness of mobile apps in training doctors in newborn care at the level of district hospitals. Android based App- ‘AIIMS-WHO CC STPs’ has easy accessibility (which is true for most Apps), is user friendly, can be updated easily (as the new guidelines evolve), has potential to be used as a point of care tool, and can be shared easily in addition to its use as a training tool for the management of sick newborns as experienced in the workshop.

The physicians had better pass percentage in knowledge scores than the skill scores. This is an interesting and a pertinent observation, and stresses the need for clinical demonstration of respective skills, return demonstration, and an ongoing training. We noted a significant improvement in the skill scores at all stations except the drug preparation station. The possible reason attributed to the difference in the skill scores could be the inadequacy of the drug preparation methodology from the workshop content.

It was found that the post-test pass percentage was lower in skill assessment than the knowledge scores. Possibly, the physicians did not have adequate time or opportunity to see the video clips another time after seeing them in the workshop. On the other hand, they had the opportunity to practice the App algorithmic contents in different clinical situations in their routine practice. We however, did not collect specific information or feedback on this aspect.

There has been a continued emphasis on competency-driven approach and information technology [29], and simple avenues like text messaging have proven to be a cost-effective learning method for health care professionals [30]. The present study supports the same approach, as the trainees reported that the App is wonderful bedside tool for management of sick newborn in health care facilities, in addition to being a good effective training tool. Once downloaded, this ‘App’ runs without the requirement of an internet connection (except the videos which are linked via YouTube) while, on tablets with storage (SD) card (which contains pre-loaded videos), these work offline as well.

This ‘App’ app shall has potential for better translation of clinical protocols and guidelines, and may decrease the time for initiating standard treatment, in addition to possibility of increased adherence to algorithmic management of clinical conditions. This ‘App’ will provide knowledge base while the skill acquisition requires both supervision and hand-on training. In addition, it is possible to monitor the usage with the help of number of downloads in different geographical areas; however, the actual usage for management of different clinical conditions and the performance in clinical scenario needs to be evaluated in another study.

The potential strengths of our study include its ‘mixed method approach’; comprising of quantitative as well as qualitative methods for evaluation. The post training assessment was done at 3 weeks, with an aim to decrease the possible spill over of pre-test and also the workshop content. The participants were medical officers from randomly chosen 25 of the total 32 districts in Tamil Nadu. These medical officers working in SNCU usually do not have any back-up support in emergency situations.

Our study has some limitations. Firstly, we evaluated only short term knowledge and skill retention in medical officers. Evaluation of knowledge and skills in medical officers after a period of atleast 3 months or even later would have been a representative of skills and knowledge retained over time. Secondly, we focused on only four modules rather than covering all newborn conditions included in the ‘AIIMS-WHO CC STPs’ App during the workshop due to time constraint. Thirdly, the current study assessed the skills by using the simulation based OSCE rather than real bedside case management by doctors while managing sick neonates.

## Conclusion

We conclude that the training using Android based App- ‘AIIMS-WHO CC STPs’ leads to an improvement in knowledge and skills among physicians working in special newborn care units in district hospitals. The trainees had confidence that the app could be used as a job aid to improve clinical practices in managing common newborn conditions. This app could also be used for refresher training of doctors.

### Supporting data

All the supporting data is included as tables and figures (attached).

## Abbreviations

SNCU: Special Newborn Care Unit
MCQ: Multiple choice questions
OSCE: Objective Structured Clinical Examination
MM: maximum marks
IQR: Interquartile range
NBSU: Newborn stabilization unit
NBCC: Newborn care corner
AIIMS: All India Institute of Medical Sciences
STP: standard treatment protocols
